# Effects of Trehalose and Galacto-Oligosaccharides on Freeze-Drying Survival and Storage Stability of *Bifidobacterium longum* B18

**DOI:** 10.3390/foods15213853

**Published:** 2026-10-28

**Authors:** Lili Liang, Lanyan Huang, Xiuhua Lin, Yongcheng Lu, Fei Yao, Shu Chen, Weipeng Guo, Qingping Wu, Ling Chen

**Affiliations:** 1 College of Food Science, South China Agricultural University, Guangzhou 510642, China; lililiang9302@126.com; 2 State Key Laboratory of Applied Microbiology Southern China, Guangdong Provincial Key Laboratory of Microbial Safety and Health, National Health Commission Science and Technology Innovation Platform for Nutrition and Safety of Microbial Food, Key Laboratory of Big Data Technologies for Food Microbiological Safety, State Administration for Market Regulation, Institute of Microbiology, Guangdong Academy of Sciences, Guangzhou 510070, China; 3 Food and Drug Laboratory, Guangdong Detection Centre of Microbiology, Guangzhou 510070, China

**Keywords:** *Bifidobacterium longum* B18, freeze-drying, trehalose, galacto-oligosaccharides, storage stability, probiotic ingredient

## Abstract

Maintaining the viability of *Bifidobacterium longum* B18 throughout lyophilization and storage remains a key challenge for its application as a probiotic ingredient in functional foods. This study screened 18 lyoprotectants, optimized trehalose (TRE) and galacto-oligosaccharide (GOS) concentrations, and evaluated post-drying survival, enzyme activities, membrane integrity, morphology, and 90-day storage stability. The 8% (*w*/*v*) TRE + 3% (*w*/*v*) GOS formulation yielded the highest post-freeze-drying survival (90.6%). It also showed higher post-drying Na^+^/K^+^-ATPase, Ca^2+^/Mg^2+^-ATPase, lactate dehydrogenase, and β-galactosidase activities than the PBS control, although responses relative to the individual protectants varied by enzyme. Qualitative fluorescence and scanning electron microscopy observations indicated limited propidium iodide-positive staining and less apparent surface damage. During storage, viability decreased in all formulations. Based on descriptive comparisons, viability retention was numerically greater at 4 and −20 °C than at 25 °C. After 90 days, the combined formulation retained 49.4% and 45.7% survival at −20 and 4 °C, respectively, compared with 38.8% and 35.8% for TRE and 30.2% and 20.3% for GOS. At 25 °C, survival decreased to 0.1%. However, because the formulations differed in total added lyoprotectant concentration, the observed differences do not establish complementary or synergistic protection by TRE and GOS. These findings support further evaluation of 8% TRE + 3% GOS for refrigerated or frozen functional foods. However, this preliminary 90-day study does not establish commercial shelf-life suitability, which requires validation in representative food matrices under commercial processing, packaging, and longer-term storage conditions.

## 1. Introduction

*Bifidobacterium longum* is a well-recognized probiotic species with documented benefits for host health, including modulation of the gut microbiota [1,2], reinforcement of intestinal barrier function [3,4], production of short-chain fatty acids (SCFAs) [5], and regulation of immune responses, with potential implications for intestinal homeostasis and neurobehavioral regulation [6,7]. Our previous study demonstrated that *B. longum* B18 has the potential to improve sleep [8] and alleviate sleep deprivation-induced anxiety-like behavior. For the industrial development of such functional strains, maintaining cell viability and functional performance during processing and storage is a critical prerequisite.

Probiotic cultures are frequently processed into dry powders by lyophilization, which removes water under low-temperature conditions and facilitates their storage and subsequent incorporation into functional foods and dietary supplements [9]. However, the process also imposes multiple stresses on probiotic cells, including freezing [10,11], dehydration [12], and oxidative damage [13], which may markedly reduce cell viability and impair subsequent functionality. In addition, viability loss may continue during storage, further limiting the practical application of freeze-dried probiotic powders [14]. Therefore, the development of appropriate lyoprotectant formulations is essential for improving survival during freeze-drying and maintaining cell viability and functional recovery during subsequent storage and rehydration.

The effectiveness of freeze-drying protectants is generally strain-dependent because probiotic strains differ in cell wall architecture, membrane composition, intracellular metabolism, and stress response capacity, resulting in distinct sensitivities to processing-related stress [15,16,17]. Moreover, the protective effects of lyoprotectants are likely to involve multiple, formulation-dependent mechanisms, including mitigation of osmotic stress, restriction of ice-crystal growth, formation of an amorphous glassy matrix, and preservation of membrane and protein integrity [18,19,20]. Accordingly, the selection of lyoprotectants for probiotic preparations usually requires strain-specific evaluation, and combinational strategies are often adopted to achieve improved protective efficacy. Among the available candidates, trehalose (TRE) and galacto-oligosaccharides (GOS) are of particular interest as food-compatible lyoprotectants. Trehalose is commonly incorporated into lyoprotective formulations because it helps maintain membrane integrity and protein stability under freezing and dehydration stresses [21,22], whereas GOS may contribute to dehydration protection and may also provide functional advantages associated with its prebiotic properties [23]. Previous studies have evaluated TRE-containing formulations for bifidobacterial preservation. Celik and O’Sullivan reported almost no viability loss during freeze-drying of *B. animalis* subsp. *lactis* Bb-12 and *B. longum* DJO10A using a formulation containing TRE and skim milk [24]. Yang et al. reported 53.22% survival of *B. longum* BIOMA 5920 with 10% TRE alone and 88.23% with an optimized formulation containing 11.50% TRE, 1.23% human-like collagen, and 4.65% glycerol [25]. GOS has also been incorporated into bifidobacterial freeze-drying formulations: Shamekhi et al. reported 62.16% survival of *B. animalis* subsp. *lactis* DSM 10140 with an optimized formulation containing skim milk and a prebiotic mixture of GOS and fructo-oligosaccharides [26].

Collectively, these studies support the use of TRE- and GOS-containing formulations. However, GOS was not evaluated alone in the cited study, and, to our knowledge, the comparative performance of TRE alone, GOS alone, and a TRE + GOS formulation under identical freeze-drying conditions has not been examined in *B. longum* B18. Therefore, a strain-specific comparison of these treatments is warranted.

A strain-specific evaluation linking formulation selection with post-freeze-drying cellular characteristics and subsequent storage viability is therefore needed. Viable-cell counts directly reflect culturability but do not capture all cellular alterations associated with freeze-drying. Measurements of selected enzyme activities, membrane-integrity staining, and morphological observations can provide complementary information on functional and structural changes. Moreover, a formulation that produces high survival immediately after freeze-drying does not necessarily provide the greatest viability retention during storage [27]. Integrating these endpoints can therefore provide a broader biological assessment of lyoprotectant performance.

In this study, 18 candidate lyoprotectants were initially screened for their protective effects on *B. longum* B18 during freeze-drying. TRE and GOS were subsequently selected for concentration optimization, and their combined formulation was further evaluated. In addition to post-freeze-drying survival, the effects of the selected formulations were assessed by measuring Na^+^/K^+^-ATPase, Ca^2+^/Mg^2+^-ATPase, lactate dehydrogenase, and β-galactosidase activities; examining membrane integrity using live/dead fluorescence staining; and observing cellular morphology by scanning electron microscopy. Viability retention of B18 in formulations containing TRE, GOS, or their combination was determined over 90 days of storage at −20, 4, and 25 °C. Building on these previous studies, the principal novelty of the present work lies in the strain-specific evaluation of *B. longum* B18 through an integrated assessment of post-freeze-drying survival, selected biochemical activities, qualitative microscopic observations, and viability retention during 90 days of storage. The overall aim was to identify an effective lyoprotectant formulation for *B. longum* B18 and to characterize its effects on post-freeze-drying survival and cellular integrity, as well as viability retention during subsequent storage, thereby providing a basis for its further development as a probiotic ingredient for refrigerated or frozen functional foods.

## 2. Materials and Methods

### 2.1. Activation and Cultivation of B. longum B18

*B. longum* B18 was originally isolated from the faeces of a healthy 3-year-old child in Xinyang, Henan, China, identified by 16S rRNA gene sequencing and BLAST analysis (https://blast.ncbi.nlm.nih.gov/Blast.cgi; accessed on 19 October 2026), and deposited at the Guangdong Microbial Culture Collection Center (GDMCC No. 67383). The strain was revived from a stock culture by streaking it onto modified de Man–Rogosa–Sharpe (mMRS) agar (composition provided in Supplementary Table S1), followed by incubation in an anaerobic workstation at 37 °C for 24–36 h. A single well-isolated colony was subsequently restreaked onto fresh mMRS agar and incubated under the same conditions. After the second passage, a single colony was aseptically inoculated into mMRS broth and incubated under the same anaerobic conditions at 37 °C until the late exponential phase (approximately 24 h) to prepare the seed culture.

### 2.2. Screening and Evaluation of the Freeze-Drying Protective System for B. longum B18

*Bifidobacterium longum* B18 cells in the late exponential growth phase were harvested by centrifugation, washed three times with sterile 0.85% (*w*/*v*) saline, and separately resuspended in 18 different lyoprotectant solutions: lactulose (99%), inulin, maltodextrin (BR), sucrose (99.9%), glucose (99%), mannitol (99%), monosodium glutamate (BR, 99%), resistant dextrin, fructose (99%), maltose (99%), sorbitol (BR, 99%), galacto-oligosaccharides (BR, 98%), trehalose (95%), skim milk (BR), arabinogalactan (90%), cellobiose (98%), xylitol (99.5%), and mannose (99%). Each prepared at 8% (*w*/*v*). All solutions were initially tested at 8% (*w*/*v*) to enable a standardized comparison and identify candidates for subsequent concentration optimization, rather than to determine the optimal concentration of each protectant. Inulin was obtained from Sigma-Aldrich (St. Louis, MO, USA); maltodextrin, monosodium glutamate, sorbitol, skim milk, and arabinogalactan were obtained from Shanghai Yuanye Bio-Technology Co., Ltd. (Shanghai, China); and the other lyoprotectants, except resistant dextrin, were obtained from Shanghai Macklin Biochemical Technology Co., Ltd. (Shanghai, China). Cells resuspended in PBS (pH 7.4; Gibco, Thermo Fisher Scientific, Waltham, MA, USA; Cat. No. 10010023; composition and molar concentrations are provided in Supplementary Table S11) served as the PBS control and were subjected to the same freeze-drying procedure. For all treatments, the ratio of wet cell pellet mass (g) to lyoprotectant solution volume (mL) was maintained at 1:3.

The resulting cell suspensions were dispensed into sterile 2-mL polypropylene screw-cap cryovials (0.4 mL per vial). The cryovials were placed on a tray inside the drying chamber, and the screw caps were removed during freeze-drying to allow water vapor to escape. The samples were pre-frozen at −80 °C for 8 h and subsequently freeze-dried for 36 h using a FreeZone freeze dryer (Labconco Corporation, Kansas City, MO, USA). During freeze-drying, the chamber pressure was maintained at 0.04 mbar, the condenser temperature was −84 °C. Operationally, drying was considered complete at the end of the predefined 36-h cycle, at which time no visible ice or frost remained in the samples. Immediately after removal from the drying chamber, the cryovials were tightly capped.

Based on the initial screening at 8% (*w*/*v*), TRE and GOS, which yielded the highest and second-highest survival rates, respectively, were selected for separate concentration screening. TRE was tested at 5%, 8%, 10%, and 12% (*w*/*v*), whereas GOS was tested at 1%, 3%, 5%, 8%, and 10% (*w*/*v*). For each protectant, the concentration yielding the highest post-freeze-drying survival within the tested range was selected. The selected concentrations were subsequently evaluated individually and as a single candidate combination containing 8% (*w*/*v*) TRE + 3% (*w*/*v*) GOS. This candidate combination was assembled from the best-performing tested concentration of each protectant.

Immediately before freeze-drying, an aliquot of each cell suspension was collected for viable-cell enumeration. After freeze-drying, the entire contents of each vial were reconstituted with sterile 0.85% (*w*/*v*) saline to the original pre-freezing volume of 0.4 mL. The pre- and post-freeze-drying suspensions were serially diluted tenfold from 10^−1^ to 10^−10^ in sterile 0.85% (*w*/*v*) saline. Aliquots (100 μL) of appropriate dilutions were spread-plated in duplicate onto mMRS agar (composition provided in Supplementary Table S1) and incubated in an anaerobic workstation (85% N_2_, 10% H_2_, and 5% CO_2_) at 37 °C for 36 h. For each independent experiment, the colony counts from the two technical replicate plates were averaged. Plates containing 30–300 colonies were used for viable-cell enumeration. Based on the lowest dilution plated (10^−1^) and a plating volume of 100 μL, the theoretical limit of detection of the plate-count method was 100 CFU/mL. All freeze-drying experiments were performed independently three times (*n* = 3). The post-freeze-drying survival rate was calculated as follows:

$$\mathrm{Lyophilized}\;\mathrm{Survival}\;\mathrm{Rate}\;=\;\frac{A_{0}}{A_{1}}\times \text{100\%}$$

where A_1_ and A_0_ denote the viable counts of the strain before and after freeze-drying, respectively, expressed as CFU.

### 2.3. Determination of Intracellular Enzyme Activities

Freeze-dried *B. longum* B18 powders were rehydrated in sterile saline (0.85% NaCl) and immediately washed three times with the sterile saline, with centrifugation at 5000× *g* for 10 min after each wash. The washed cell pellets were resuspended in cell lysis buffer and disrupted on ice using an ultrasonic cell disruptor (Sonics & Materials, Inc., Newtown, CT, USA). Following centrifugation at 15,000× *g* for 10 min at 4 °C, the resulting supernatants were used to determine Na^+^/K^+^-ATPase (catalog no. BC0065), Ca^2+^/Mg^2+^-ATPase (catalog no. BC0960), lactate dehydrogenase (LDH; catalog no. BC0685), and β-galactosidase (catalog no. BC2585) activities using commercial assay kits (Beijing Solarbio Science & Technology Co., Ltd., Beijing, China) according to the manufacturer’s instructions. All four kit manuals include bacterial sample preparation by sonication followed by centrifugation, supporting their intended use with bacterial lysates. Total protein concentrations were determined using the BCA assay, and enzyme activities were normalized to the total protein concentration and expressed as U mg^−1^ protein.

### 2.4. Scanning Electron Microscopy Analysis of Freeze-Dried Bacterial Cell Morphology

Freeze-dried *B. longum* B18 powder was resuspended in sterile saline, washed, and centrifuged to collect the cells for scanning electron microscopy (SEM) analysis. Cells collected before freeze-drying were processed in parallel as the pre-freeze-drying control. All samples were fixed overnight in 2.5% glutaraldehyde at 4 °C. The samples were subsequently dehydrated through a graded ethanol series (30%, 50%, 70%, 80%, 90%, and 100%), treated with tert-butanol, and freeze-dried. Before imaging, the samples were sputter-coated with gold, and micrographs were acquired using an Apreo 2S HiVac field-emission scanning electron microscope (Thermo Fisher Scientific, Brno, Czech Republic) to evaluate changes in cell morphology [28]. SEM observations were performed on three independent biological preparations per treatment, with five microscopic fields examined per preparation. The SEM micrographs were acquired at an accelerating voltage of 3.0 kV, a beam current of 25 pA, and a magnification of 20,000× using the T2 detector, with working distances of approximately 9–11 mm. Cell morphology was assessed qualitatively, and no quantitative morphometric analysis was performed. After reviewing all examined fields, an assessor blinded to treatment identity selected representative micrographs from each coded group based on image quality and morphological representativeness.

### 2.5. Assessment of Membrane Integrity of Freeze-Dried Bifidobacterium longum B18 by CLSM

Approximately 500 mg of freeze-dried *B. longum* B18 powder was resuspended in 2 mL of sterile 0.85% NaCl solution, followed by centrifugation and two washes with the same solution. SYTO 9/PI staining was performed using a bacterial viability assay kit (L7012) for qualitative CLSM observations. Briefly, 3 μL of SYTO 9 and 1 μL of propidium iodide were mixed thoroughly, and 4 μL of the mixed dye solution was added to 2 mL of the cell suspension. After incubation in the dark for 25 min, the suspension was centrifuged, and the resulting pellet was resuspended in 200 μL of NaCl solution. Fluorescence images were acquired using a confocal laser scanning microscope (CLSM; LSM 800, Carl Zeiss AG, Oberkochen, Germany). CLSM observations were performed on three independent biological preparations per treatment, with five microscopic fields examined per preparation.

### 2.6. Effect of Different Storage Temperatures on the Stability of Freeze-Dried B. longum B18

Freeze-dried *B. longum* B18 powders prepared with 8% TRE + 3% GOS, 8% TRE, or 3% GOS were stored at 4, −20, or 25 °C for 90 days. Samples were analyzed before storage (day 0) and subsequently at 10-day intervals (days 10–90). The powders were retained in the original 2-mL polypropylene screw-cap cryovials, each containing the powder obtained from 0.4 mL of pre-freezing cell suspension. The screw caps were tightly closed and additionally sealed with laboratory sealing film. No vacuum sealing or gas flushing was used. Samples were protected from light, and a separate, previously unopened vial was used at each sampling time. Relative humidity during storage was not measured. At each sampling point, 0.025 g of freeze-dried powder was suspended in 0.225 mL of sterile 0.85% (*w*/*v*) saline to prepare an initial 10^−1^ suspension. The suspension was serially diluted tenfold, and 100 μL aliquots of appropriate dilutions were spread-plated in duplicate following the plate-count procedure described in Section 2.2. For each independent experiment, colony counts from the two technical replicate plates were averaged, and viable cell counts were expressed as CFU/g of freeze-dried powder. The storage experiment was performed independently three times (n = 3). Viability retention during storage was expressed relative to the viable count measured on day 0 according to the following equation:

$$\mathrm{Viability}\;\mathrm{retention}\;=\;\frac{N_{t}}{N_{0}}\times \text{100\%}$$

where *N*_0_ and *N*_t_ denote the viable cell counts of the freeze-dried powder on day 0 and after t days of storage, respectively, expressed as CFU/g.

## 3. Statistical Analysis

Data are reported as mean ± standard deviation (SD). Statistical processing was carried out in GraphPad Prism 10.12 (GraphPad Software, Boston, MA, USA). Normality was assessed using the Shapiro–Wilk test, and homogeneity of variance was assessed using the Brown–Forsythe test. For comparisons involving three or more groups, normally distributed data satisfying the assumption of homogeneity of variance were analyzed by one-way ANOVA with Tukey’s post hoc multiple-comparison test. When the normality assumption was not met, differences among groups were evaluated using the Kruskal–Wallis test with Dunn’s post hoc procedure. Statistical significance was defined as *p* < 0.05. For the one-way ANOVA analyses, Tukey-adjusted *p*-values and simultaneous 95% confidence intervals for the pairwise mean differences are provided in Supplementary Tables S3–S10. The 90-day storage data were summarized descriptively as the mean ± SD of three independently prepared and stored samples (*n* = 3); no inferential statistical comparisons were performed.

## 4. Results

### 4.1. Effects of Lyoprotectant Type, Concentration, and Combined Formulation on Post-Lyophilization Viability of B. longum B18

**Figure 1:**
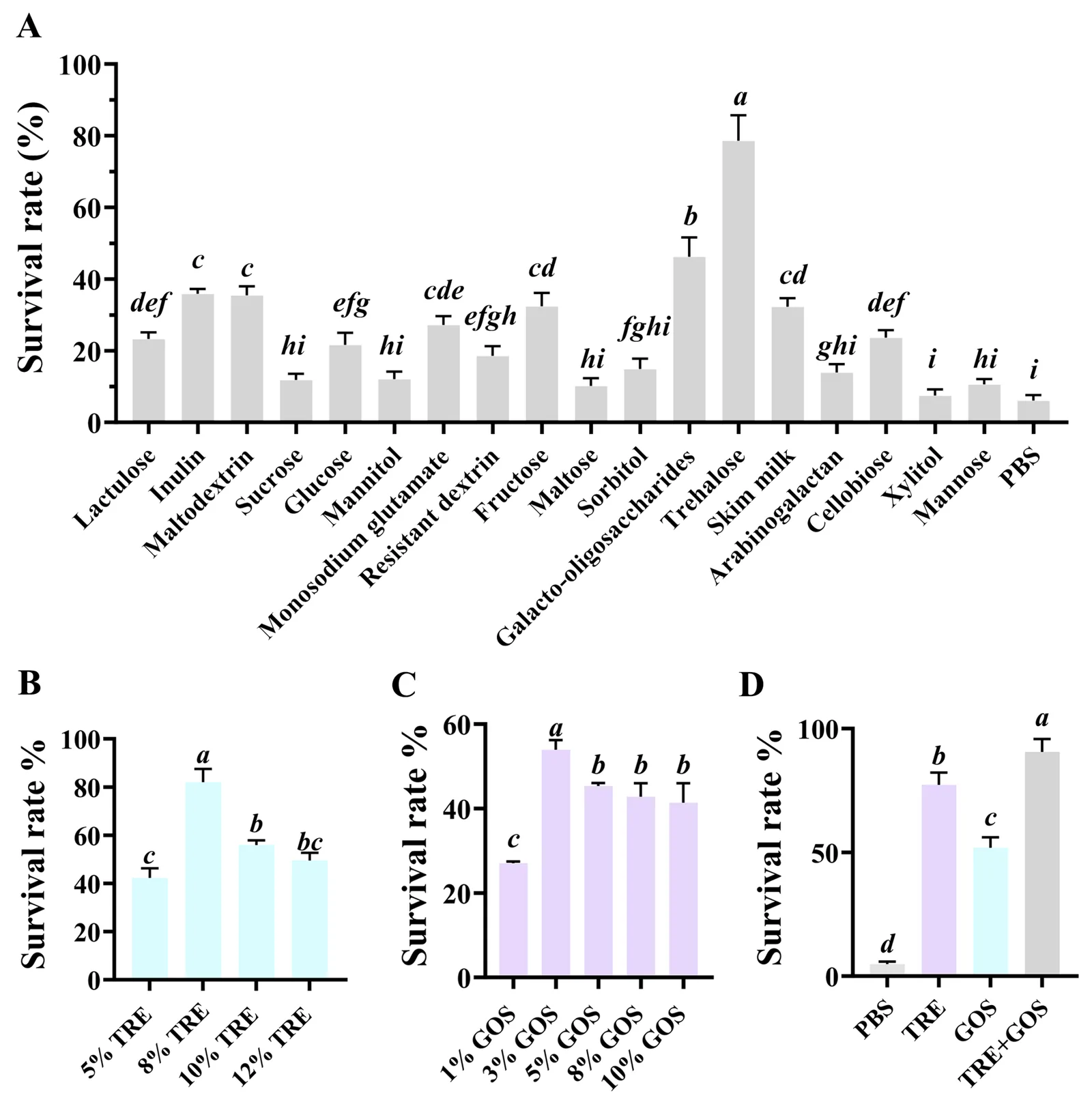
Effects of lyoprotectant type, concentration, and combined formulation on the post-freeze-drying survival of *B. longum* B18. (**A**) Screening of different lyoprotectants; (**B**) effects of different trehalose concentrations; (**C**) effects of different galacto-oligosaccharide concentrations; and (**D**) effects of the selected lyoprotectants used individually and in combination. Panels (**A**–**D**) were generated from separate experiments. For each treatment, data are presented as the mean ± SD from three independent freeze-drying experiments (*n* = 3). Data within each panel were analyzed separately using one-way ANOVA followed by Tukey’s multiple comparisons test. Different lowercase letters within the same panel indicate significant differences among treatments (*p* < 0.05), whereas groups sharing at least one letter are not significantly different.

At an initial concentration of 8% (*w*/*v*), the candidate lyoprotectants were compared on the basis of *B. longum* B18 survival after lyophilization. Survival rates differed markedly among the tested protectants, indicating a clear dependence on lyoprotectant type (Figure 1A). Under the initial screening conditions, trehalose (TRE) was the best-performing candidate, yielding a survival rate of 78.6%, followed by galacto-oligosaccharides (GOS) at 46.2%, whereas inulin, maltodextrin and skim milk resulted in survival rates of 35.9%, 35.5% and 32.3%, respectively. Based on these findings, TRE and GOS were further subjected to concentration optimization using four and five concentration levels, respectively. Survival rates varied with protectant concentration (Figure 1B,C). Within the tested ranges, the highest survival rates were obtained with 8% (*w*/*v*) TRE and 3% (*w*/*v*) GOS, reaching 82.0% and 53.9%, respectively. These concentrations were subsequently evaluated individually and in combination (Figure 1D). The survival rate of the 8% TRE + 3% GOS formulation was 90.6%, which was significantly higher than those of the individual TRE and GOS formulations (*p* < 0.05; Figure 1D). However, because the formulations differed in total lyoprotectant concentration, this comparison does not establish complementary or synergistic protection by TRE and GOS.

### 4.2. Effects of Different Lyoprotectants on the Intracellular Key Enzyme Activities of Freeze-Dried B. longum B18

**Figure 2:**
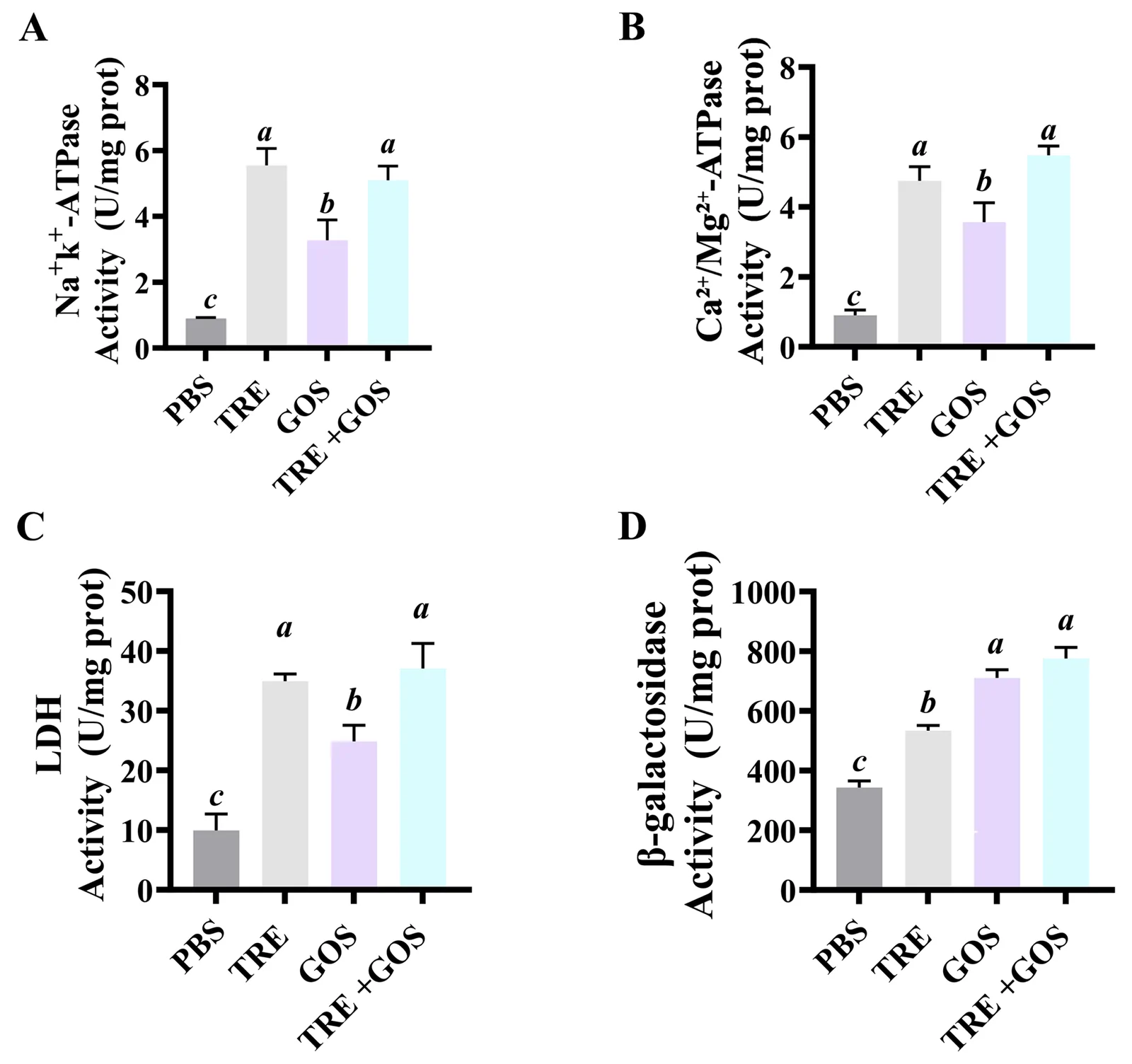
Effects of different lyoprotectants on selected cellular enzyme activities of *B. longum* B18 after freeze-drying. (**A**) Na^+^/K^+^-ATPase activity; (**B**) Ca^2+^/Mg^2+^-ATPase activity; (**C**) LDH activity; and (**D**) β-galactosidase activity. Data are presented as the mean ± SD (n = 3). Different lowercase letters indicate significant differences among groups, whereas groups sharing the same letter are not significantly different (one-way ANOVA followed by Tukey’s multiple-comparisons test, *p* < 0.05).

The activities of the selected enzymes in freeze-dried *B. longum* B18 varied among the four lyoprotectant treatments (Figure 2). Na^+^/K^+^-ATPase, Ca^2+^/Mg^2+^-ATPase, and LDH activities were significantly higher in the 8% TRE and 8% TRE + 3% GOS groups than in the 3% GOS and PBS groups (Figure 2A–C; *p* < 0.05). No significant differences in the activities of these three enzymes were observed between the 8% TRE and 8% TRE + 3% GOS groups (*p* > 0.05). In contrast, β-galactosidase activities in the 3% GOS and 8% TRE + 3% GOS groups were 710.62 and 775.67 U mg^−1^ protein, respectively, with no significant difference between the two groups (Figure 2D; *p* > 0.05). Both groups exhibited significantly higher β-galactosidase activities than the 8% TRE group (533.87 U mg^−1^ protein) and the PBS group (343.05 U mg^−1^ protein). Furthermore, β-galactosidase activity in the 8% TRE group was significantly higher than that in the PBS group (*p* < 0.05).

### 4.3. Effects of Different Lyoprotectants on the Cell Membrane Integrity of B. longum B18

**Figure 3:**
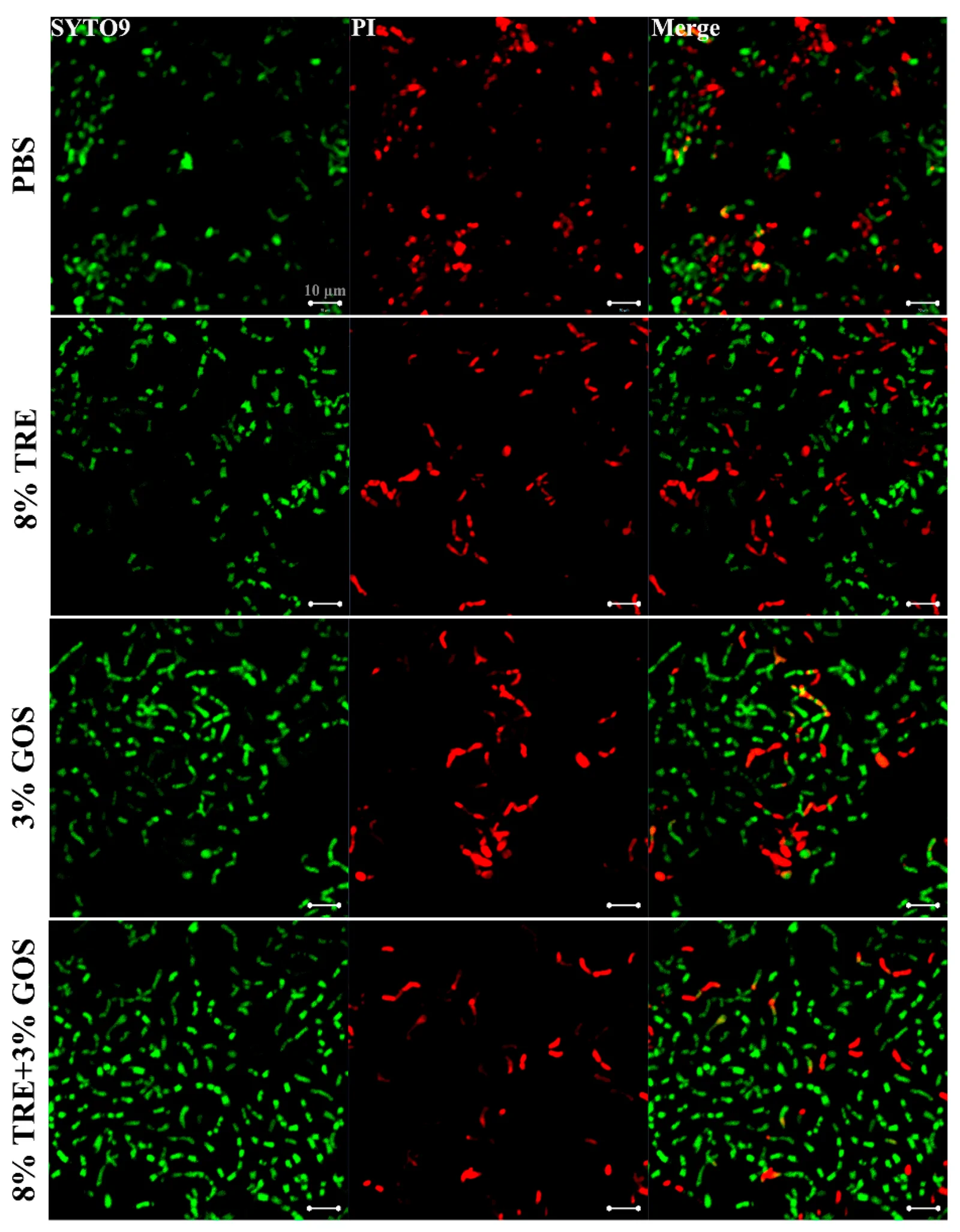
Representative confocal laser scanning microscopy (CLSM) images showing SYTO 9/PI staining patterns in *B. longum* B18 after freeze-drying with different lyoprotectant formulations. SYTO 9 (green) labels cells with both intact and compromised membranes, whereas propidium iodide (PI; red) preferentially stains cells with compromised membranes. The columns show the SYTO 9, PI, and merged fluorescence images, respectively, whereas the rows represent the PBS control, 8% (*w*/*v*) TRE, 3% (*w*/*v*) GOS, and 8% (*w*/*v*) TRE + 3% (*w*/*v*) GOS groups. Scale bars = 10 µm.

SYTO 9/PI fluorescence staining was used to qualitatively examine *B. longum* B18 after freeze-drying. SYTO 9 labels cells with both intact and compromised membranes, whereas PI preferentially stains cells with compromised membranes. Representative confocal laser scanning microscopy (CLSM) images are shown in Figure 3. In the displayed fields, the PBS group showed more prominent PI-positive staining, whereas less prominent PI-positive staining was observed in the 8% TRE and 3% GOS groups. The 8% TRE + 3% GOS group showed predominantly SYTO 9-positive staining with comparatively limited PI-positive staining. These qualitative observations do not independently establish differences in membrane integrity or viable-cell fractions among formulations. In addition, because the formulations differed in total lyoprotectant concentration, the staining pattern observed in the representative images of the combined group cannot be attributed specifically to a complementary or synergistic interaction between TRE and GOS.

### 4.4. SEM Analysis of the Morphology of Freeze-Dried B. longum B18 Cells

**Figure 4:**
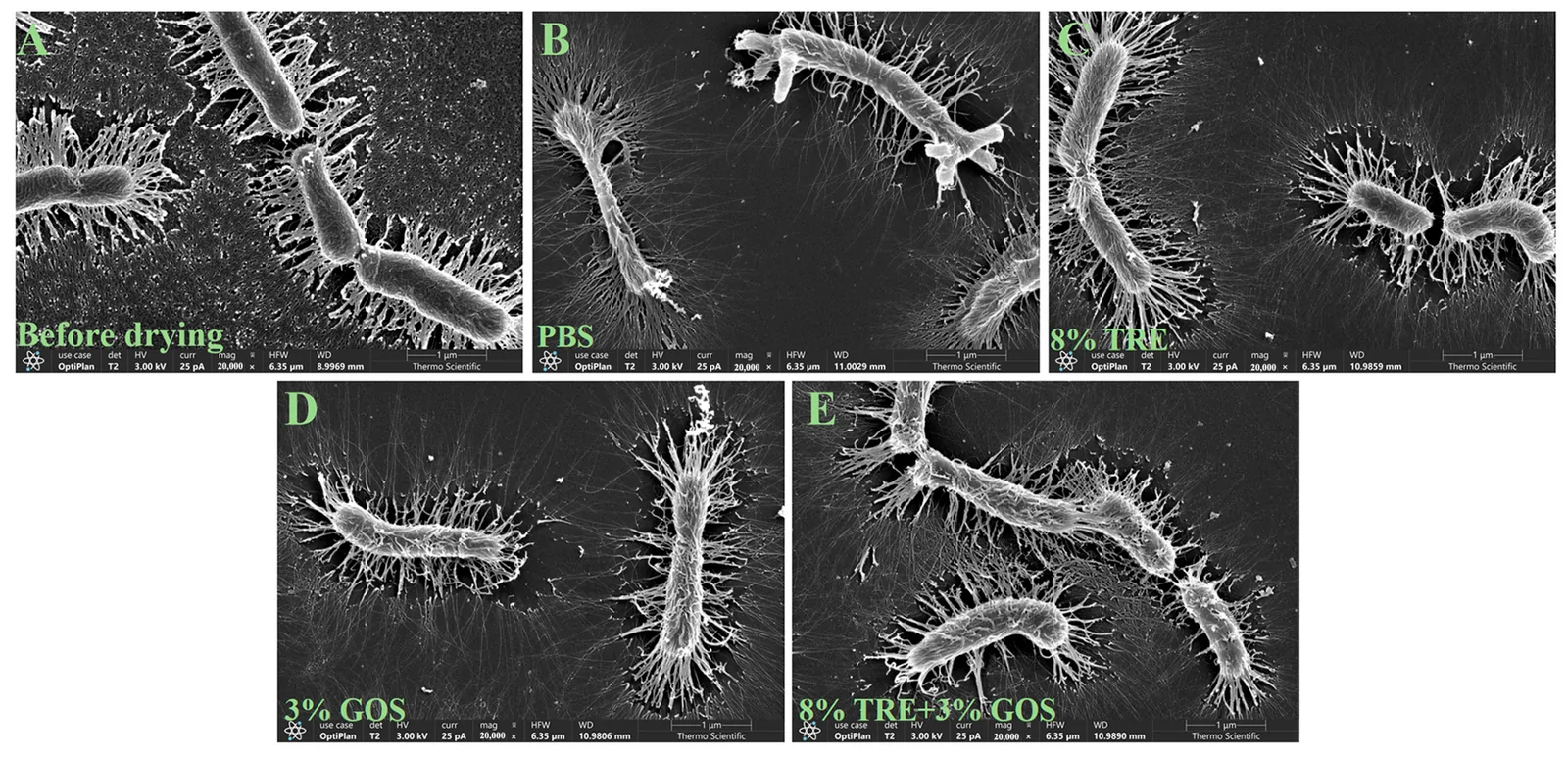
Representative scanning electron micrographs of B18 before and after freeze-drying under different formulation conditions. (**A**) Cells before freeze-drying; (**B**) PBS control; (**C**) 8% (*w*/*v*) TRE; (**D**) 3% (*w*/*v*) GOS; and (**E**) 8% (*w*/*v*) TRE combined with 3% (*w*/*v*) GOS. PBS served as the unprotected control. Scale bars =1 µm.

Representative SEM micrographs are shown in Figure 4. Cells in the pre-freeze-drying control exhibited an intact rod-shaped morphology with clear contours. After freeze-drying, cells in the PBS group appeared markedly shrunken and deformed, with visible surface collapse. In contrast, cells prepared with 8% TRE or 3% GOS generally retained a more recognizable rod-shaped morphology, although varying degrees of surface deformation remained visible. Cells in the 8% TRE + 3% GOS group appeared comparatively intact, with clear contours and less apparent surface deformation, more closely resembling the pre-freeze-drying control. These observations are qualitative and may be influenced by morphological artifacts introduced during SEM sample preparation, including fixation, dehydration, and drying. Potential complementary or synergistic effects of TRE and GOS remain unresolved because total lyoprotectant concentrations differed among formulations.

### 4.5. Storage Stability of Freeze-Dried B. longum B18 Powder

**Figure 5:**
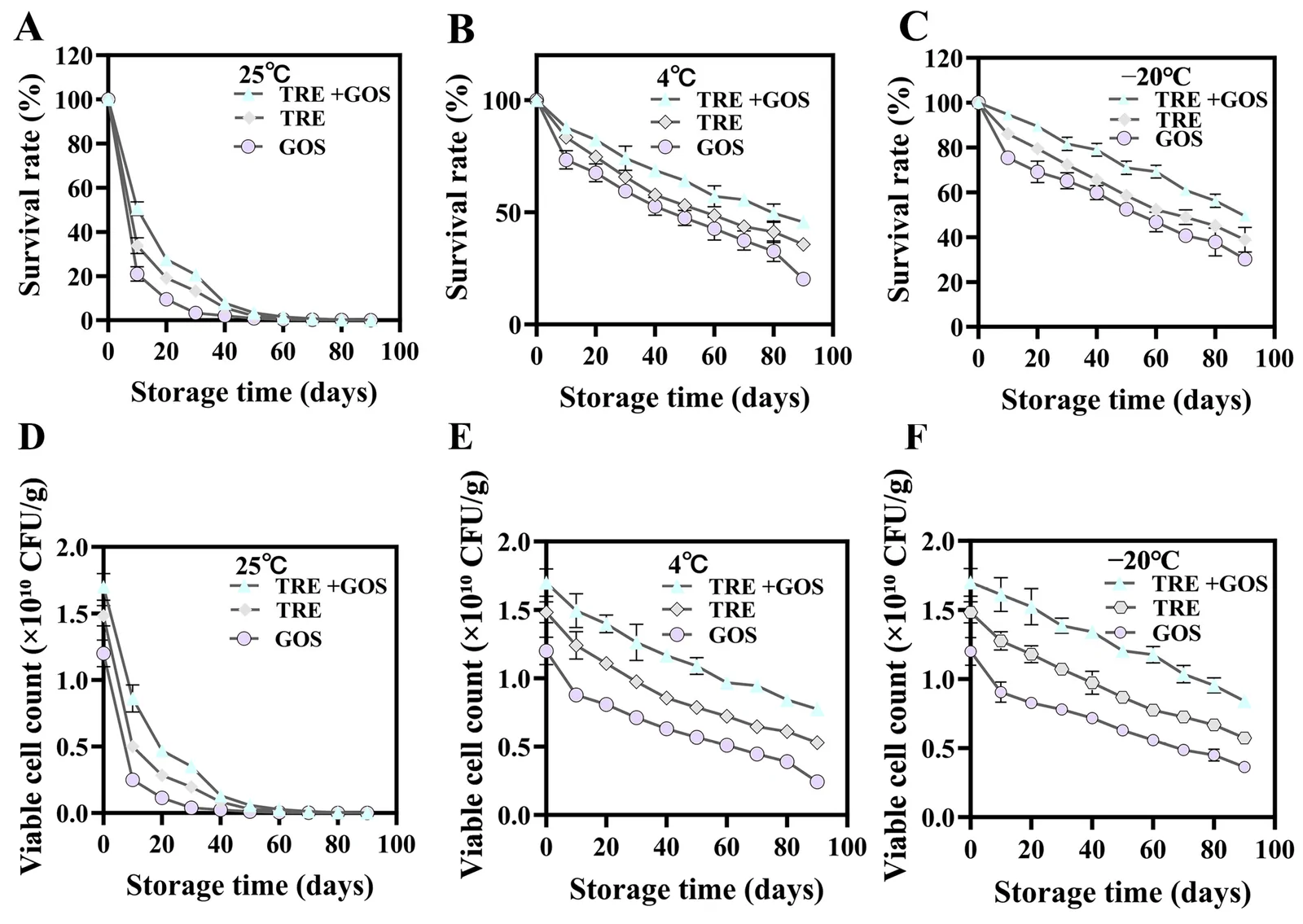
Effects of lyoprotectant formulation and storage temperature on the stability of freeze-dried *B. longum* B18 over 90 days. (**A**–**C**) Survival rates normalized to the respective day 0 values and (**D**–**F**) viable cell counts of freeze-dried powders stored at (**A**,**D**) 25 °C, (**B**,**E**) 4 °C, and (**C**,**F**) −20 °C. The powders were prepared with a combination of 8% (*w*/*v*) TRE and 3% (*w*/*v*) GOS (TRE + GOS), 8% (*w*/*v*) TRE, or 3% (*w*/*v*) GOS. Data are presented descriptively as the mean ± SD of three independently prepared and stored samples (*n* = 3); no inferential statistical comparisons were performed.

The mean viable cell counts at day 0 were 1.7 × 10^10^, 1.48 × 10^10^, and 1.2 × 10^10^ CFU/g for the TRE + GOS, TRE, and GOS formulations, respectively (Figure 5D–F). Both the survival rates, calculated relative to the respective day 0 values, and the viable cell counts decreased progressively during the 90-day storage period, with more rapid declines observed at 25 °C than at 4 or −20 °C (Figure 5). From day 10 onward, the TRE + GOS formulation showed the numerically highest survival rate at each storage temperature, followed by the TRE and GOS formulations. After 90 days at −20 °C, the survival rates of the TRE + GOS, TRE, and GOS formulations were 49.4%, 38.8%, and 30.2%, respectively, corresponding to viable cell counts of 8.4 × 10^9^, 5.7 × 10^9^, and 3.6 × 10^9^ CFU/g. At 4 °C, the corresponding survival rates were 45.7%, 35.8%, and 20.3%, with viable cell counts of 7.8 × 10^9^, 5.3 × 10^9^, and 2.4 × 10^9^ CFU/g, respectively. At 25 °C, the survival rates decreased sharply during the first 10 days to 50.1%, 33.8%, and 20.7% for the TRE + GOS, TRE, and GOS formulations, respectively. After 90 days at 25 °C, the survival rates were only 0.11%, 0.06%, and 0.06%, corresponding to viable cell counts of 1.87 × 10^7^, 9.4 × 10^6^, and 7.3 × 10^6^ CFU/g, respectively. Overall, the TRE + GOS formulation showed the highest numerical survival rate and viable cell count among the formulations tested at each storage temperature, while viability was maintained to a greater extent at 4 and −20 °C than at 25 °C.

## 5. Discussion

The present study demonstrated that the protective efficacy of lyoprotectants for *Bifidobacterium longum* B18 depended on both the type and concentration of the protectant. Initial screening at 8% (*w*/*v*) revealed marked differences among the tested lyoprotectants, with TRE maintaining the highest B18 viability after lyophilization, GOS showing a moderate effect, and inulin and maltodextrin providing comparatively limited protection. Subsequent optimization identified 8% TRE and 3% GOS as the best-performing concentrations among those tested, resulting in survival rates of 82.0% and 53.9%, respectively. Combining 8% TRE with 3% GOS further increased the survival rate to 90.6%, representing the highest value among the formulations tested. The enzyme-activity measurements, fluorescence staining, morphological observations, and storage experiment collectively indicated that the combined formulation exhibited favorable overall performance. However, the differences observed during storage were numerical and were not statistically evaluated. However, because the combined formulation contained a higher total lyoprotectant concentration than either single-protectant formulation, the present design cannot determine whether its higher observed survival was associated with formulation composition, total lyoprotectant concentration, or both. Accordingly, these results do not establish a complementary or synergistic interaction between TRE and GOS.

Skim milk is widely used as an effective lyoprotectant for bifidobacteria because its proteins and lactose can help stabilize cellular structures during freezing, drying, and rehydration. However, 8% (*w*/*v*) skim milk provided only moderate protection for *B. longum* B18 in the present study. This result may be associated with the fixed concentration used in the initial screening, as previous studies commonly employed higher skim milk concentrations, as well as the strain-specific response of B18 and differences in freeze-drying and rehydration conditions. These findings indicate that 8% skim milk was not among the best-performing protectants for B18 under the standardized 8% (*w*/*v*) screening condition.

The responses of B18 to TRE and GOS were clearly concentration-dependent but not monotonic. B18 survival increased from 42.3% at 5% TRE to 82.0% at 8% TRE; however, at higher TRE concentrations of 10% and 12%, survival decreased to 55.9% and 49.5%, respectively. A similar pattern was observed for GOS, as survival rose from 27.1% at 1% GOS to 53.9% at 3% GOS, before gradually decreasing at concentrations between 5% and 10%. These findings indicate that each carbohydrate had a best-performing concentration within the tested range rather than providing protection in direct proportion to its concentration. A related study on *B. longum* Reuter 1963 also showed that lyoprotectant performance depended on formulation concentration; the highest numerical survival among the maltodextrin treatments was obtained at 75% relative to bacterial dry matter, whereas increasing the concentration to 100% provided no further improvement, and trehalose at 100% bacterial dry matter was not protective [29]. Direct comparison should nevertheless be made with caution because the concentration basis and freeze-drying conditions differed from those used in the present study. At concentrations below the best-performing tested levels, the amount of protectant may have been insufficient to adequately stabilize cellular components or establish an effective amorphous matrix during dehydration, consistent with the water-replacement and vitrification mechanisms proposed for protective sugars [30]. However, increasing TRE or GOS above these levels did not provide additional protection and instead reduced survival. One possible explanation is that the higher solids content may have altered ice formation and water removal during freeze-drying, which could in turn have influenced the residual moisture content and structural properties of the dried matrix [31,32,33]. Previous studies using GOS- and FOS-based matrices have likewise demonstrated that carbohydrate composition and the physical state of the dried matrix influence bacterial preservation during drying and storage [34,35,36]. Nevertheless, the present study did not determine the residual moisture content, water activity, or glass-transition temperature of the dried powders. Therefore, the mechanism responsible for the decrease in survival above 8% TRE and 3% GOS cannot be established from the current data. These findings demonstrate that a higher protectant concentration does not necessarily result in greater protection and highlight the need to optimize lyoprotectant concentrations for each bacterial strain and freeze-drying process.

Of particular relevance to the combined-formulation comparison, survival decreased from 82.0% at 8% TRE to 55.9% and 49.5% at 10% and 12% TRE, respectively. Thus, within the single-protectant TRE series, increasing the total protectant concentration did not produce a monotonic improvement in survival. This finding makes the 90.6% survival observed with the 11% total-solids TRE + GOS formulation noteworthy and argues against a simple assumption that more solids necessarily provide greater protection. Nevertheless, the non-monotonic TRE response does not remove the confounding between composition and total concentration and does not establish an interaction between TRE and GOS. Because 11% TRE, 11% GOS, and alternative TRE:GOS ratios at 11% total solids were not evaluated under the same conditions, the present study cannot distinguish a composition effect from a total-concentration effect. Equal-total-solids controls, including 11% TRE, 11% GOS, and at least one additional TRE:GOS ratio such as 5.5% TRE + 5.5% GOS, together with a fixed-total-solids mixture or factorial design, will be needed to determine whether an optimal TRE:GOS ratio exists.

Trehalose is widely recognized as an effective lyoprotectant that helps preserve membrane integrity and protein stability under freezing and dehydration conditions, possibly through water substitution and hydrogen-bond interactions that may reduce membrane damage, protein denaturation, and aggregation [21,37]. Previous work in *Lactiplantibacillus plantarum* showed that trehalose increased freeze-drying survival by 3.15-fold and LDH activity by 2.47-fold after lyophilization [20]. Consistent with these findings, both TRE-containing formulations showed significantly higher post-freeze-drying Na^+^/K^+^-ATPase, Ca^2+^/Mg^2+^-ATPase, and LDH activities than the GOS and PBS treatments. The ATP-hydrolyzing activities were measured using commercial kits designated “Na^+^/K^+^-ATPase” and “Ca^2+^/Mg^2+^-ATPase” and reflect ATP hydrolysis under the respective kit-defined conditions, without identifying specific ATPase proteins or demonstrating ion-transport capacity. Because all activities were normalized to total lysate protein, potentially including contributions from non-viable cells, these results characterize selected biochemical activities in bulk lysates rather than enzyme activities per viable cell or preservation of overall cellular metabolism. None of the three activities differed significantly between TRE and TRE + GOS. These measurements therefore do not explain the observed survival difference between the two formulations or establish a specific TRE–GOS interaction. GOS has been proposed to support protection during drying by contributing to glass formation, helping maintain residual water around biomolecular surfaces, and stabilizing cellular structures and biomolecules [38,39]. In the present study, β-galactosidase activity was higher in the GOS and TRE + GOS groups than in the TRE and PBS groups, whereas Na^+^/K^+^-ATPase, Ca^2+^/Mg^2+^-ATPase, and LDH activities were higher in the TRE-containing formulations. β-Galactosidase is not a neutral marker of post-freeze-drying viability because its expression in *B. longum* is influenced by carbon source and strain-specific regulation, including carbon catabolite repression [40]. The higher activity in the GOS-containing formulations could therefore reflect preservation of pre-existing enzyme, carbon-source-related regulation, or both. Because β-galactosidase expression and GOS consumption were not measured, these results demonstrate neither GOS-induced enzyme synthesis nor GOS metabolism by B18 and should not be interpreted as a direct measure of post-drying survival. Nevertheless, the β-galactosidase activity detected after freeze-drying may be technologically relevant to refrigerated dairy applications because of its potential roles in lactose hydrolysis and in situ GOS production [40]. However, these functions, including activity at refrigeration temperatures, require validation in B18 and dairy matrices. Notably, although the TRE + GOS formulation achieved the highest survival rate, this advantage was not accompanied by uniformly higher activities across all four enzymes, suggesting that no single measured enzyme activity accounted for its overall performance. This interpretation is also consistent with the qualitative CLSM and SEM observations. However, because protein conformation, membrane lipid organization, and protectant–cell interactions were not directly examined, the proposed mechanisms remain inferential.

The fluorescence and SEM observations were also consistent with the protective effects of the selected lyoprotectant formulations. In the representative images, The PBS group displayed more prominent PI-positive staining, whereas comparatively less PI-positive staining was observed in the TRE and GOS groups. The TRE + GOS group showed predominantly SYTO 9-positive cells with relatively limited PI-positive staining. Similarly, SEM revealed pronounced shrinkage and surface deformation in cells from the PBS control, whereas cells prepared with TRE or GOS generally retained a more recognizable rod-shaped morphology. Among the lyoprotectant-treated groups, cells in the combined TRE + GOS formulation appeared comparatively intact, with clear contours and less apparent surface deformation. Collectively, these qualitative observations suggest that the combined formulation was associated with less apparent morphological damage during freeze-drying, consistent with its higher post-freeze-drying survival rate. However, because the formulations differed in total lyoprotectant concentration, the apparently better preservation observed with the combined formulation cannot be attributed specifically to a complementary or synergistic interaction between TRE and GOS.

The practical applicability of freeze-dried probiotic preparations depends largely on the preservation of cell viability throughout storage and distribution [35]. Because the formulations differed in added solids, absolute CFU/g comparisons may be influenced by differences in final powder mass and should not be interpreted solely as differences in cell survival. Absolute CFU/g values describe viable-cell content per gram of final powder, while storage stability was assessed primarily using viability retention relative to each formulation’s own post-freeze-drying day 0 baseline. In the present study, the survival rates of all three formulations progressively decreased during the 90-day storage period, with numerically more rapid declines observed at 25 °C than at 4 or −20 °C. After 90 days of storage at −20 °C, the survival rates were 49.4%, 38.8%, and 30.2% for the TRE + GOS, TRE, and GOS formulations, respectively. The corresponding values at 4 °C were 45.7%, 35.8%, and 20.3%. Thus, at day 90, the TRE + GOS formulation showed the highest numerical survival rate at both temperatures among the formulations tested. At 25 °C, survival in all three formulations decreased to ≤0.11% after 90 days, indicating poor viability retention during prolonged ambient-temperature storage under the tested conditions. Trehalose-containing formulations have similarly been reported to improve the storage stability of freeze-dried *Lactobacillus plantarum* LP90 [41]. The ability of sugars to restrict molecular mobility in an amorphous glassy state has been associated with slower membrane deterioration, protein inactivation, and other degradation reactions during storage [16,20,42], while loss of glassy-state stability may accelerate biological inactivation [43]. Such mechanisms may partly explain the numerically greater viability retention observed for the TRE-containing formulations in the present study. However, because glass transition temperature, residual moisture content, and water activity were not measured, the involvement of glass-state stabilization remains a plausible explanation rather than a mechanism directly demonstrated by the current results.

These findings have potential implications for the development of B18 as a probiotic ingredient for functional foods. Because TRE and GOS are carbohydrates used in food applications [44,45], their use as lyoprotectants could support the future incorporation of the freeze-dried preparation into food formulations. The pronounced numerical differences in viability retention among the tested storage temperatures suggest that the current formulation warrants further evaluation for use in refrigerated or frozen products rather than in products intended for ambient-temperature distribution. Potential applications may include refrigerated dairy products, plant-based foods, and other functional food formulations maintained under cold-chain conditions. However, probiotic stability in an actual food product may differ from that observed in the model powder system because food composition, pH, water activity, oxygen exposure, processing conditions, and interactions with other ingredients can affect cell viability [46,47]. Although GOS is recognized as a prebiotic carbohydrate [48,49], the present study evaluated it primarily as a lyoprotectant and did not determine its effective dose or prebiotic function in a finished food product. Consequently, the TRE + GOS preparation should be described as a GOS-containing probiotic formulation rather than a confirmed synbiotic product [50]. Overall, the present results provide a basis for further evaluating freeze-dried B18 as a candidate probiotic ingredient in cold-chain functional foods. Validation in representative food matrices and under commercial processing, packaging, and shelf-life conditions remains necessary before product suitability and storage performance can be established.

Several limitations of the present study should be considered when interpreting the findings. First, the evaluation was restricted to a single strain, *B. longum* B18; therefore, whether the best-performing formulation identified under the tested conditions would perform similarly with other bifidobacterial strains remains to be established. Second, the combined formulation contained a higher total lyoprotectant concentration than either single-protectant formulation, so its superior performance cannot be attributed unequivocally to an interaction between TRE and GOS. Future studies should include equal-total-solids controls and use factorial or fixed-total-solids mixture designs to distinguish concentration effects from interactions between TRE and GOS. Residual moisture content, water activity, glass transition temperature, and oxygen exposure during storage were not measured, limiting mechanistic interpretation of the observed differences in storage stability. CLSM observations were qualitative, without cell-fraction quantification, measured or standardized staining cell densities, or fresh and killed B18 cells as controls. Equal powder masses did not ensure equivalent cell numbers. Potential dye interactions [51] and PI-related artefacts reported in Gram-positive biofilms [52] were not assessed in B18. Accordingly, these images provide qualitative descriptions of staining patterns, with limited scope for comparing membrane integrity or viable-cell fractions among formulations. SEM observations should also be interpreted cautiously because fixation, dehydration, and drying during sample preparation may have introduced morphological artifacts. Residual protectant, if present, could also affect apparent cell contours and surface texture, limiting morphological comparisons among treatments. Moreover, storage performance was assessed primarily on the basis of viable cell counts over 90 days, whereas post-storage acid and bile tolerance, growth recovery, metabolic activity, and other functional properties were not evaluated. Finally, because the 90-day storage data were summarized descriptively rather than analyzed using a factorial repeated-measures approach or mixed-effects model, the present study cannot establish the main or interactive effects of formulation, storage temperature, and time. Future confirmatory storage studies with adequate independent replication should employ an appropriate factorial repeated-measures or mixed-effects model to evaluate these effects. Longer-term studies incorporating physicochemical characterization and post-storage functional assessments in representative food matrices are therefore needed before the formulation’s suitability for practical applications can be established.

## 6. Conclusions

In conclusion, the performance of TRE and GOS as lyoprotectants for *B. longum* B18 depended on protectant type and concentration. Among the formulations tested, 8% (*w*/*v*) TRE + 3% (*w*/*v*) GOS yielded the highest post-freeze-drying survival rate. Compared with the PBS control, this formulation was associated with higher post-freeze-drying activities of selected enzymes and less apparent membrane and morphological damage; it also showed the numerically highest survival rates after 90 days of storage at 4 and −20 °C. However, because the combined formulation contained a higher total protectant concentration than either single-protectant formulation, the present results do not establish complementary or synergistic protection by TRE and GOS and do not demonstrate that 8% TRE + 3% GOS is an optimal combined formulation. The poor viability retention observed at 25 °C further indicates the need for low-temperature storage under the tested conditions. Overall, these preliminary 90-day laboratory data support further evaluation of the formulation for refrigerated or frozen functional foods but do not establish commercial shelf-life suitability, which requires validation in representative food matrices under commercial processing, packaging, and longer-term storage conditions.

## Data Availability

The data supporting the findings of this study are available from the corresponding author upon reasonable request.

## Supplementary Materials

The following supporting information can be downloaded at: https://www.mdpi.com/article/10.3390/foods15213853/s1, Table S1: Composition of the modified MRS (mMRS) medium used for the cultivation of *Bifidobacterium longum* B18; Table S2: Principles of the four enzyme activity assays; Table S3: Selected pairwise comparisons for lyoprotectant screening; Table S4: Pairwise comparisons among trehalose concentrations; Table S5: Pairwise comparisons among galacto-oligosaccharide concentrations; Table S6: Pairwise comparisons among lyoprotectant formulations; Table S7: Pairwise comparisons for Na^+^/K^+^-ATPase activity; Table S8: Pairwise comparisons for Ca^2+^/Mg^2+^-ATPase activity; Table S9: Pairwise comparisons for LDH activity; Table S10: Pairwise comparisons for β-galactosidase activity; Table S11: Composition and specifications of phosphate-buffered saline.
