# Supplementary material for: Effects of Trehalose and Galacto-Oligosaccharides on Freeze-Drying Survival and Storage Stability of Bifidobacterium longum B18
Source: Foods. 2026 Oct 28;15(21):3853. doi: 10.3390/foods15213853 (PMC13763081; doi:10.3390/foods15213853)
Supplement: Supplementary file 1 [file foods-15-03853-s001.zip › foods-4613139-supplementary.pdf]

Supplementary materials

1. Supplementary Results

Table S1. Composition of the modified MRS (mMRS) medium used for the cultivation of *Bifidobacterium longum* B18.

Component	Final concentration
Tryptone	10 g/L
Beef extract	10 g/L
Yeast extract	5 g/L
Glucose	20 g/L
Anhydrous sodium acetate	5 g/L
Dipotassium hydrogen phosphate trihydrate ($K_2HPO_4 \cdot 3H_2O$)	2.6 g/L
Diammonium hydrogen citrate	2 g/L
Magnesium sulfate heptahydrate ($MgSO_4 \cdot 7H_2O$)	0.1 g/L
Manganese sulfate monohydrate ($MnSO_4 \cdot H_2O$)	0.05 g/L
Tween 80	1 g/L
L-Cysteine	0.5 g/L
Agar	15 g/L

For liquid mMRS medium, the same formulation was used without agar.

Table S2. Principles of the four enzyme activity assays

Assay	Assay principle	Detection wavelength (nm)
Na^+/K^+-ATPase	ATP is hydrolyzed to ADP and inorganic phosphate under the kit assay conditions. The released inorganic phosphate is quantified using a molybdenum-blue colorimetric reaction.	660
Ca^{2+}/Mg^{2+}-ATPase	ATP is hydrolyzed to ADP and inorganic phosphate under the kit assay conditions. The released inorganic phosphate is quantified using a molybdenum-blue colorimetric reaction.	660
L-Lactate dehydrogenase	L-Lactate is oxidized to pyruvate in an NAD^+-dependent reaction. Pyruvate subsequently reacts with 2,4-dinitrophenylhydrazine to form a colored hydrazone, which is measured under alkaline conditions.	450
β-Galactosidase	p-Nitrophenyl β-D-galactopyranoside is hydrolyzed to release p-nitrophenol, which is quantified colorimetrically.	400

The assay principles were summarized from the manufacturer's instructions (Beijing Solarbio Science & Technology Co., Ltd., Beijing, China).

Table S3. Selected pairwise comparisons involving trehalose or galacto-oligosaccharides for the lyoprotectant screening experiment presented in Figure 1A.

Comparison	95% CI of the mean difference	Tukey-adjusted p-value
Trehalose vs. Lactulose	46.04 to 64.58	<0.0001
Trehalose vs. Inulin	33.44 to 51.98	<0.0001
Trehalose vs. Maltodextrin	33.88 to 52.42	<0.0001
Trehalose vs. Sucrose	57.50 to 76.04	<0.0001
Trehalose vs. Glucose	47.69 to 66.23	<0.0001
Trehalose vs. Mannitol	57.29 to 75.83	<0.0001
Trehalose vs. Monosodium glutamate	42.14 to 60.68	<0.0001
Trehalose vs. Resistant dextrin	50.81 to 69.35	<0.0001
Trehalose vs. Fructose	36.95 to 55.49	<0.0001
Trehalose vs. Maltose	59.18 to 77.72	<0.0001
Trehalose vs. Sorbitol	54.43 to 72.97	<0.0001
Trehalose vs. Galacto-oligosaccharides	23.13 to 41.67	<0.0001
Trehalose vs. Skim milk	37.10 to 55.64	<0.0001
Trehalose vs. Arabinogalactan	55.43 to 73.97	<0.0001
Trehalose vs. Cellobiose	45.7 to 64.24	<0.0001
Trehalose vs. Xylitol	61.83 to 80.37	<0.0001
Trehalose vs. Mannose	58.72 to 77.26	<0.0001
Trehalose vs. PBS	63.29 to 81.83	<0.0001
GOS vs. Lactulose	13.65 to 32.19	<0.0001
GOS vs. Inulin	1.039 to 19.58	0.0167
GOS vs. Maltodextrin	1.482 to 20.02	0.0102
GOS vs. Sucrose	25.10 to 43.64	<0.0001
GOS vs. Glucose	15.29 to 33.83	<0.0001
GOS vs. Mannitol	24.90 to 43.44	<0.0001
GOS vs. Monosodium glutamate	9.742 to 28.28	<0.0001
GOS vs. Resistant dextrin	18.41 to 36.95	<0.0001
GOS vs. Fructose	4.5560 to 23.1	0.0003
GOS vs. Maltose	26.78 to 45.32	<0.0001
GOS vs. Sorbitol	22.03 to 40.57	<0.0001
GOS vs. Skim milk	4.702 to 23.24	0.0002
GOS vs. Arabinogalactan	23.03 to 41.57	<0.0001
GOS vs. Cellobiose	13.3 to 31.84	<0.0001
GOS vs. Xylitol	29.44 to 47.98	<0.0001
GOS vs. Mannose	26.32 to 44.86	<0.0001
GOS vs. PBS	30.90 to 49.44	<0.0001

Table S4. Pairwise comparisons for the trehalose concentration experiment presented in Figure 1B.

Comparison	95% CI of the mean difference	Tukey-adjusted p-value
5% TRE vs. 8% TRE	−49.84 to −29.57	<0.0001
5% TRE vs. 10% TRE	−23.72 to −3.449	0.0113
5% TRE vs. 12% TRE	−17.35 to 2.921	0.1822
8% TRE vs. 10% TRE	15.99 to 36.25	0.0002
8% TRE vs. 12% TRE	22.36 to 42.62	<0.0001
10% TRE vs. 12% TRE	−3.765 to 16.5	0.2597

Table S5. Pairwise comparisons for the galacto-oligosaccharide concentration experiment presented in Figure 1C.

Comparison	95% CI of the mean difference	Tukey-adjusted p-value
1% GOS vs. 3% GOS	−34.22 to −19.50	<0.0001
1% GOS vs. 5% GOS	−25.65 to −10.93	<0.0001
1% GOS vs. 8% GOS	−23.10 to −8.379	0.0003
1% GOS vs. 10% GOS	−21.69 to −6.969	0.0006
3% GOS vs. 5% GOS	1.209 to 15.93	0.0216
3% GOS vs. 8% GOS	3.759 to 18.48	0.0040
3% GOS vs. 10% GOS	5.169 to 19.89	0.0016
5% GOS vs. 8% GOS	−4.811 to 9.911	0.7829
5% GOS vs. 10% GOS	−3.401 to 11.32	0.4386
8% GOS vs. 10% GOS	−5.951 to 8.771	0.9665

Table S6. Pairwise comparisons for the selected lyoprotectants used individually and in combination presented in Figure 1D.

Comparison	95% CI of the mean difference	Tukey-adjusted p-value
PBS vs. TRE	−83.40 to −61.33	<0.0001
PBS vs. GOS	−58.05 to −35.98	<0.0001
PBS vs. TRE + GOS	−96.77 to −74.70	<0.0001
TRE vs. GOS	14.31 to 36.39	0.0004
TRE vs. TRE + GOS	−24.41 to −2.333	0.0196
GOS vs. TRE + GOS	−49.76 to −27.68	<0.0001

Table S7. Pairwise comparisons for Na^+/K^+-ATPase activity presented in Figure 2A.

Comparison	95% CI of the mean difference	Tukey-adjusted p-value
PBS vs. TRE	−5.848 to −3.452	<0.0001
PBS vs. GOS	−3.568 to −1.172	0.001
PBS vs. TRE + GOS	−5.391 to −2.996	<0.0001
TRE vs. GOS	1.082 to 3.478	0.0013
TRE vs. TRE + GOS	−0.7411 to 1.654	0.6319
GOS vs. TRE + GOS	−3.021 to −0.6256	0.0054

Table S8. Pairwise comparisons for Ca^{2+}/Mg^{2+}-ATPase activity presented in Figure 2B.

Comparison	95% CI of the mean difference	Tukey-adjusted p-value
PBS vs. TRE	−4.830 to −2.857	<0.0001
PBS vs. GOS	−3.647 to −1.673	0.0001
PBS vs. TRE + GOS	−5.560 to −3.587	<0.0001
TRE vs. GOS	0.1967 to 2.170	0.0206
TRE vs. TRE + GOS	−1.717 to 0.2566	0.161
GOS vs. TRE + GOS	−2.900 to −0.9267	0.0012

Table S9. Pairwise comparisons for LDH activity presented in Figure 2C.

Comparison	95% CI of the mean difference	Tukey-adjusted p-value
PBS vs. TRE	−32.68 to −17.38	<0.0001
PBS vs. GOS	−22.59 to −7.288	0.0011
PBS vs. TRE + GOS	−34.77 to −19.47	<0.0001
TRE vs. GOS	2.441 to 17.74	0.0124
TRE vs. TRE + GOS	−9.745 to 5.552	0.8163
GOS vs. TRE + GOS	−19.84 to −4.538	0.0041

Table S10. Pairwise comparisons for β-galactosidase activity presented in Figure 2D.

Comparison	95% CI of the mean difference	Tukey-adjusted p-value
PBS vs. TRE	−261.7 to −119.9	0.0001
PBS vs. GOS	−438.5 to −296.7	<0.0001
PBS vs. TRE + GOS	−503.5 to −361.7	<0.0001
TRE vs. GOS	−247.7 to −105.9	0.0002
TRE vs. TRE + GOS	−312.7 to −170.9	<0.0001
GOS vs. TRE + GOS	−135.9 to 5.855	0.0726

Table S11. Composition and specifications of phosphate-buffered saline

Component	Chemical formula	Concentration (mg/L)	Molar concentration (mM)
Sodium chloride	$NaCl$	9000	155.172
Potassium dihydrogen phosphate	KH_2PO_4	144	1.059
Disodium hydrogen phosphate heptahydrate	$Na_2HPO_4 \cdot 7H_2O$	795	2.966
